# SARS-CoV-2 Variants and Their Relevant Mutational Profiles: Update Summer 2021

**DOI:** 10.1128/Spectrum.01096-21

**Published:** 2021-11-17

**Authors:** Mohammad Alkhatib, Valentina Svicher, Romina Salpini, Francesca Alessandra Ambrosio, Maria Concetta Bellocchi, Luca Carioti, Lorenzo Piermatteo, Rossana Scutari, Giosuè Costa, Anna Artese, Stefano Alcaro, Robert Shafer, Francesca Ceccherini-Silberstein

**Affiliations:** a Department of Experimental Medicine, University of Rome Tor Vergata, Rome, Italy; b Dipartimento di Scienze della Salute, Campus S. Venuta, Università degli Studi “Magna Graecia” di Catanzaro, Catanzaro, Italy; c Net4Science Academic Spin-Off, Campus S. Venuta, Università Magna Græcia di Catanzaro, Catanzaro, Italy; d Division of Infectious Diseases, Stanford University School of Medicine, Stanford, California, USA; University of Georgia

**Keywords:** COVID-19, emerging variants, mutations, pandemic, SARS-CoV-2, variants

## Abstract

Since the beginning of the coronavirus disease 2019 (COVID-19) pandemic caused by it, severe acute respiratory syndrome coronavirus 2 (SARS-CoV-2) has been undergoing a genetic diversification leading to the emergence of new variants. Nevertheless, a clear definition of the genetic signatures underlying the circulating variants is still missing. Here, we provide a comprehensive insight into mutational profiles characterizing each SARS-CoV-2 variant, focusing on spike mutations known to modulate viral infectivity and/or antigenicity. We focused on variants and on specific relevant mutations reported by GISAID, Nextstrain, Outbreak.info, Pango, and Stanford database websites that were associated with any clinical/diagnostic impact, according to published manuscripts. Furthermore, 1,223,338 full-length high-quality SARS-CoV-2 genome sequences were retrieved from GISAID and used to accurately define the specific mutational patterns in each variant. Finally, mutations were mapped on the three-dimensional structure of the SARS-CoV-2 spike protein to assess their localization in the different spike domains. Overall, this review sheds light and assists in defining the genetic signatures characterizing the currently circulating variants and their clinical relevance.

**IMPORTANCE** Since the emergence of SARS-CoV-2, several recurrent mutations, particularly in the spike protein, arose during human-to-human transmission or spillover events between humans and animals, generating distinct worrisome variants of concern (VOCs) or of interest (VOIs), designated as such due to their clinical and diagnostic impacts. Characterizing these variants and their related mutations is important in tracking SAR-CoV-2 evolution and understanding the efficacy of vaccines and therapeutics based on monoclonal antibodies, convalescent-phase sera, and direct antivirals. Our study provides a comprehensive survey of the mutational profiles characterizing the important SARS-CoV-2 variants, focusing on spike mutations and highlighting other protein mutations.

## INTRODUCTION

Severe acute respiratory syndrome coronavirus 2 (SARS-CoV-2), the new member of the coronavirus family that emerged in December 2019 and caused the coronavirus disease 2019 (COVID-19) pandemic, is characterized by a large genome with a length of 29,891 nucleotides, encoding 4 structural proteins and 16 nonstructural proteins (NSP) with regulatory functions. In order to guarantee a proper replication of its complex genome, SARS-CoV-2, like the other coronavirus members, is endowed with a higher replication fidelity than other RNA viruses, which is ensured by an exoribonuclease protein (NSP.14-ExoN) that allows proofreading function and, in turn, limits the accumulation of mutations (1, 2).

Nevertheless, SARS-CoV-2 is undergoing a process of genetic diversification, consistent with adaptation to humans fueled by the massive circulation of the virus worldwide in a short time (causing more than 227 million infections to date [GISAID, 16 September 2021]) (3). This has led to a progressive increase in the SARS-CoV-2 evolutionary rate over time. Indeed, a previous study, led in the first semester of 2020, estimated an evolution rate of around 2/10,000 nucleotides/year (4). More recent estimates have shown that the SARS-CoV-2 evolutionary rate has undergone a substantial increase to 6.6/1,000 nucleotides/year (5). In accordance with this diversification, there are a growing number of studies describing the emergence of new SARS-CoV-2 clades and variants. In just over 1 year of the SARS-CoV-2 pandemic, over 20,000 distinct viral mutations, including several insertions/deletions, have been reported across the viral genome (5). A subset of these variants have been reported to increase viral transmission, such as Alpha B.1.1.7 (5, 6) and Delta B.1.617.2 (7, 8), while others have been reported to escape humoral immunity (Beta B.1.351 and Gamma P.1) (9–11). Among the different viral proteins, the spike glycoprotein has so far been characterized by the faster accumulation of mutations due to its critical role in mediating viral infectivity and antigenicity (12, 13). In particular, this protein is composed of the S1 subunit (residues 1 to 690), containing the receptor binding domain (RBD; residues 319 to 541) and several epitopes recognized by neutralizing antibodies, and the S2 subunit (residues 691 to 1273), promoting fusion between the viral envelope and the plasma membrane of the host cell (14). Mutations in the spike glycoprotein have raised global concerns for their association with enhanced transmissibility, greater disease severity, risk of reinfection, potential diagnostic impact, and decreased vaccine efficacy (15, 16).

**FIG 1:**
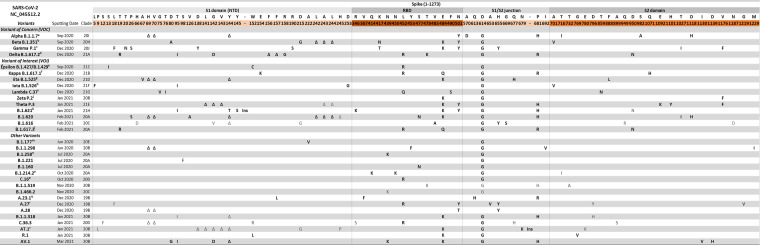
Mutations underlying the currently circulating variants in the spike glycoprotein. Only mutated positions are reported. The different domains of the spike glycoproteins are depicted. The consensus sequence for each variant was defined as nonsynonymous substitutions or deletions that occurred in >75% of sequences within that lineage. Each mutation (such as E484K) is indicated by a first letter that is the symbol for the reference amino acid of NC_045512.2 (e.g., E), a number for the amino acid position in the wild-type protein (e.g., 484), and a second letter representing the amino acid actually found in the sequence analyzed (e.g., K). The nomenclatures of the VOCs and some of the VOIs were those reported by WHO and Pango, while the rest of the VOIs and other variants were reported by Pango. Mutations in black refer to the mutations reported by Nextstrain, Outbreak.info, Pango lineages, and Stanford database websites, while mutations in gray are those that we identified by analyzing entire high-quality viral genome sequences from GISAID (*n* = 1,223,383). ^a^ The mutations L452R, E484K, and S494L are rarely present in this variant, with rates of 0.05%, 0.3%, and 0.3%, respectively. In addition to the deletion at position 144, a deletion at position 145 is also observed, with a low prevalence of about 0.02%. ^b^ This VOC was previously characterized additionally by the presence of L18F, which currently is only in about 38% of sequences, and 2 sublineages have evolved recently (B.1.351.2 and B.1.351.3) that have L18F at prevalences of about 94% and 93%, respectively. ^c^ The mutation P681H is rarely present in this variant, with a prevalence of 1.3%. ^d^ The mutations V70F, A222V, W258L, and K417N are detected in this variant with prevalences of about 0.3%, 12.1%, 0.2%, and 0.3%, respectively. Recently, this variant has evolved into 3 sublineages (AY.1, AY.2, and AY.3) that have acquired some additional mutations, as follows: AY.1 (also called Delta plus) presents W258L and K417N and AY.2 presents A222V and K417N, while AY.3 does not present specific Spike mutations. ^e^ The mutations S13I and W152C are only present in the B.1.429 variant. ^f^ The mutations T19I, G142D, and H1101D are detected in this variant with prevalences of 54.5%, 71.3%, and 30.8%, respectively. ^g^ The mutation Q52R is detected in this variant with a prevalence of 71.6%. ^h^ The mutations L452R, S477N, and E484K cooccur rarely in this variant, while they have sole prevalences of about 25.7%, 15.1%, and 54.0%, respectively. Recently, the B.1.526 variant has evolved into 2 sublineages (B.1.526.1 and B.1.526.2) that appear to have several more unique mutations. B.1.526.1 presents the mutations D80G, Y144Δ, F157S, L452R, D614G, T859N, and D950H, while B.1.526.2 presents L5F, T95I, D253G, S477N, D614G, and Q957R. ^i^ A large deletion of 7 amino acids between residues 247 and 253 is detected in 63.6% of sequences of this variant. ^j^ The mutation F565L is detected in this variant with a prevalence of about 6.9%. ^k^ An insertion is present at 145/146N in all sequences. ^l^ The mutation G142D is detected in this variant with a prevalence of 43.8%. ^m^ The mutations A262S and P272L can be detected with prevalences of 7.5% and 6.1%, respectively. ^n^ The deletion at positions 69 and 70 is detected in about 71% of sequences. ^o^ A 3-amino-acid insertion at 214TDR is present at a prevalence of 71.3% in this variant. ^p^ The mutations S98F, G769V, and K854N are detected with prevalences of 2.9%, 32.5%, and 8.9%, respectively. ^q^ The mutations R102I, E484K, and P812S are detected in this variant with prevalences of 50.5%, 6.0%, and 5.3%, respectively. ^r^ The mutation Q677H is present with a prevalence of about 28.1%. ^s^ A large deletion of 9 amino acids at residues 136 to 144 and an insertion of 4 amino acids at 679GIAL are present in all sequences.

A variant of concern (VOC) is a variant for which there is evidence of an increase in transmissibility, increase in disease severity (increased hospitalizations or deaths), significant reduction in neutralization by antibodies generated during previous infection or vaccination, reduced effectiveness of treatments or vaccines, or diagnostic detection failures (16, 17) (https://www.who.int/en/activities/tracking-SARS-CoV-2-variants/). Based on their epidemiological characteristics and patterns of spike mutations, four variants have been classified as VOCs by the WHO, U.S. Centers for Disease Control and Prevention (CDC), or European Centre for Disease Prevention and Control (ECDC). These variants, including B.1.1.7, B.1.351, P.1, and B.1.617.2 (recently renamed by WHO as Alpha, Beta, Gamma, and Delta, respectively) (Fig. 1), were first identified in the United Kingdom, South Africa, Brazil, and India, respectively, and currently are the predominant strains in several other countries (16, 17).

Alpha B.1.1.7 was the first VOC, identified in September 2020 for its higher transmissibility and increased pathogenicity (18). The Beta B.1.351 and Gamma P.1 VOCs have raised global concerns because of their association with an increased risk of reinfection and/or reduced vaccine efficacy (particularly for Beta B.1.351) (9, 10), likely related to an altered antigenicity associated with the E484K mutation (a change of E to K at position 484) (16, 17). The recent Delta B.1.617.2 is still raising concern due to its high transmissibility, immune escape capability, and risk of reinfection (7, 8). Furthermore, beyond these 4 VOCs, 11 additional variants of interest (VOIs) have been identified, whose role in hampering the control of the pandemic still needs to be better elucidated (16, 17). VOIs, characterized by specific mutations in biologically important regions, are increasing in prevalence, but evidence for their increased transmissibility, virulence, and/or diagnostics, therapeutics, or immune escape is still lacking. Furthermore, a VOI is usually associated with an increased proportion of cases or unique outbreak clusters, with limited prevalence or expansion only in selected countries (16, 17). Beyond the above-mentioned VOCs and VOIs, another 17 variants have been identified that are characterized by mutations associated with increased viral transmissibility or infectivity or altered antigenicity (3, 18–21).

Notably, the currently identified variants are undergoing a further genetic diversification with the accumulation of novel mutations and generation of novel variants like the Alpha B.1.1.7 and A.23.1 variants, which have further genetic signatures in the spike glycoprotein, including E484K (22) and E484K along with Q613H (23), respectively. Interestingly, the recently identified Delta B.1.617.2 VOC has already evolved into three sublineages, defined as AY.1 (Delta plus), AY.2, and AY.3. Both of the sublineages AY.1 and AY.2 have acquired the K417N mutation, as well as specific mutations like W258L for AY.1 and A222V for AY.2, while AY.3 is defined by the specific mutation I162V in NS6, whose biological significance is not yet known (16).

Here, we provide a comprehensive overview of the mutations characterizing each VOC and VOI and other variants that have not formally been classified into one of these categories, with an emphasis on their roles in modulating spike protein function and antigenicity.

## RESULTS AND DISCUSSION

### SARS-CoV-2 variants and their relevant mutational characterization.

**FIG 2:**
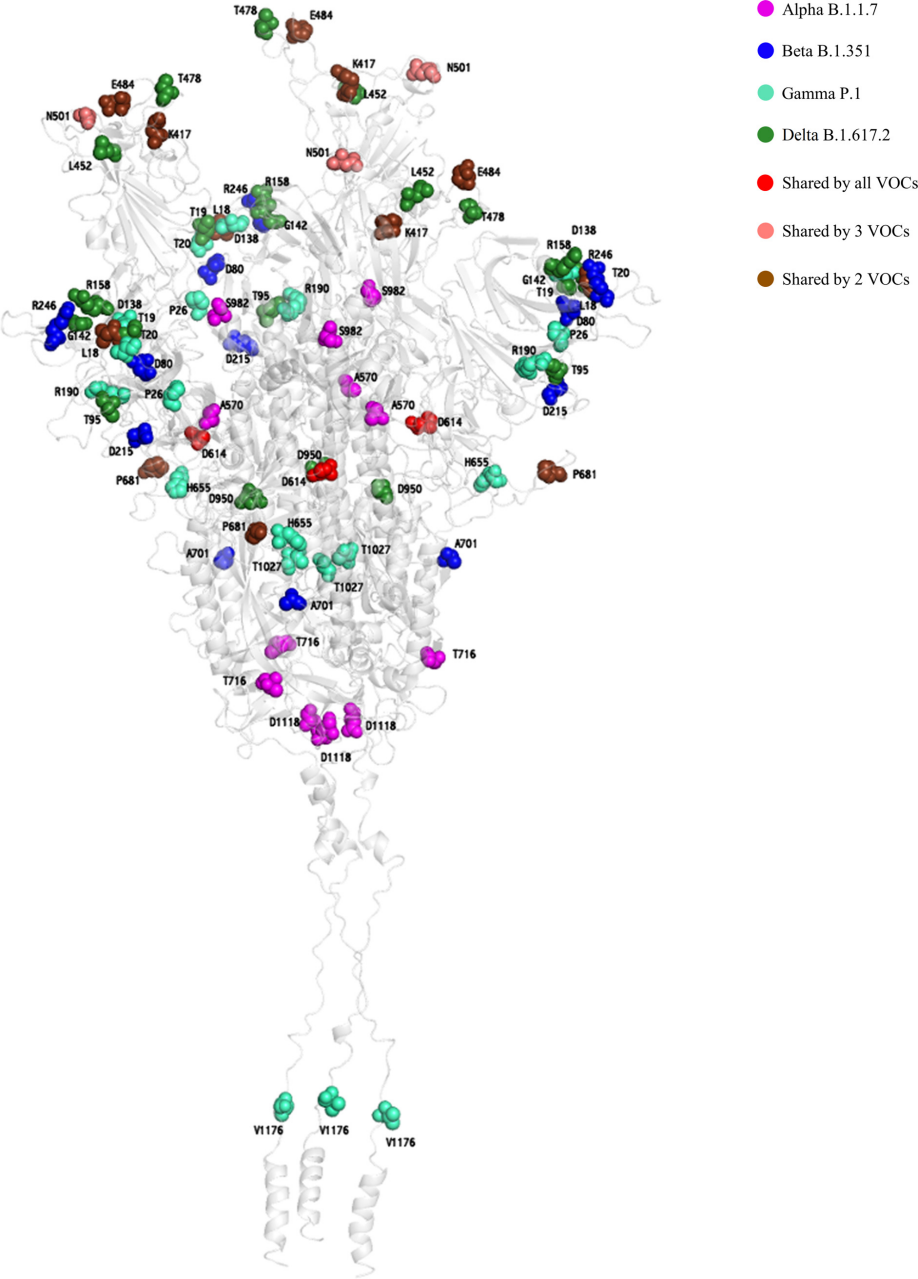
Three-dimensional representation of SARS-CoV-2 spike protein reporting residues characterizing the 4 variants of concern (VOCs). The protein is shown as a gray cartoon. The Alpha B.1.1.7, Beta B.1.351, Gamma P1, and Delta B.1.617.2 VOCs are represented as magenta, blue, cyan and forest-green spheres, respectively. The shared mutated residues present in all, 3, and 2 VOCs are reported as red, salmon, and chocolate spheres, respectively.

Figure 1 shows an alignment of spike amino acid mutations for each of the WHO/CDC/ECDC VOCs, VOIs, and other variants. The alignment is divided into four parts: the N-terminal domain (NTD), receptor-binding domain (RBD), S1/S2 junction, and S2. Figure 2 depicts the locations of the most commonly occurring mutations in VOCs within the context of the three-dimensional structure of the trimer spike protein. The attention is focused on key mutations in subunit 1 of the spike glycoprotein (each representing the consensus amino acid for a specific variant), since they are under extensive investigation for their roles in modulating viral infectivity and antigenicity (Fig. 1). Generally, such mutations occur in multiple variants. Conversely, less functional characterization is available for mutations in subunit 2, which are characterized by sporadic presence in a single variant (with the exception of D950N and V1176F). It is noteworthy that the results presented on the functional characterization of spike mutations are mainly based on the use of spike-expressing pseudoviruses exposed to soluble ACE2 receptor or to a different spectrum of monoclonal antibodies, convalescent plasma, and plasma from vaccinated individuals, as a consequence of the limited availability of high-level biosafety laboratories (Table 1). This model permits specifically addressing the role in the entry process without fully recapitulating all the steps of the SARS-CoV-2 life cycle.

**TABLE 1:** Mutations present in the currently circulating variants and their functional characterization

Mutation(s) or deletion(s) characterizing SARS-CoV-2 variants	Variant(s)^*a*^	Location^*b*^	Potential impact^*c*^						
			Increase in:			Escape from:			
			Infectivity^*d*^	Transmissibility^*e*^	Disease severity^*f*^	Single or multiple antibodies	Convalescent sera	Vaccine	Diagnostic assay detection
Mutations									
S13I	Epsilon	NTD	NA	NA	NA	Yes	NA	NA	NA
L18F	Gamma, A.27	NTD	NA	NA	NA	Yes	NA	NA	NA
T20N	Gamma	NTD	NA	NA	NA	Yes	NA	NA	NA
D80A, D80G	Beta, AV.1	NTD	NA	NA	NA	Yes	NA	NA	NA
W152C, W152L, W152R	Epsilon, R.1, C.36.3	NTD	NA	NA	NA	Yes	NA	NA	NA
D215G	Beta, B.1.616, AT.1	NTD	NA	NA	NA	Yes	NA	NA	NA
A222V	B.1.177	NTD	No	Yes	No	No	No	No	No
D253G	Iota	NTD	NA	NA	NA	Yes	NA	NA	NA
V367F	A.23.1	RBD	Yes	NA	NA	Yes	NA	NA	NA
K417N, K417T	Beta, Gamma	RBD	No	No	No	Yes	Yes	Yes	No
N439K	B.1.258, B.1.466.2, AV.1	RBD	Yes	No	No	Yes	Yes	No	No
L452R, L452Q	Delta, Epsilon, Kappa, B.1.617.3, C.16, A.27, C36.3, Lambda	RDB	Yes	No	No	Yes	Yes	No	No
Y453F	B.1.1.298	RBD	Yes	No	No	Yes	No	No	No
S477N	B.1.160, B.1.620	RBD	Yes	Yes	No	Yes	Yes	Yes	No
T478K	Delta, B.1.1.519	RBD	NA	NA	NA	Yes	NA	NA	NA
V483A	B.1.616	RDB	NA	NA	NA	Yes	NA	NA	NA
E484K, E484Q	Beta, Gamma, Eta, Zeta, Theta, B.1.621, B.1.620, B.1.1.318, AT.1, R.1, AV.1, Kappa, B.1.617.3	RBD	No	No	No	Yes	Yes	Yes	No
N501Y	Alpha, Beta, Gamma, Theta, B.1.621, A.27, A.28	RBD	Yes	Yes	No	Yes	Yes	Yes	No
D614G	All variants except A.23.1, A.27, and A.28	S1/S2	Yes	Yes	No	No	No	No	No
Q677H	Eta, C.36.3	S1/S2	NA	NA	No	No	No	No	No
P681H, P681R	Alpha, Theta, B.1.621, B.1.620, B.1.1.519, B.1.1.318, AV.1, Kappa, Delta, B.1.617.3, A.23.1	S1/S2	Yes	NA	No	No	No	No	No

Deletions									
Del H69-V70	Alpha, Eta, B.1.620, B.1.1.298, A.28, C.36.3	NTD	Yes	No	No	Yes	No	No	Yes
Del L141-G142-V143	Theta	NTD	No	No	No	Yes	No	No	No
Del Y144	Alpha, Eta, B.1.620, B.1.616, B.1.1.318, AV.1	NTD	No	No	No	Yes	No	No	No
Del L242-A243-L244	Beta, Theta, B.1.620	NTD	No	No	No	Yes	No	No	No

a The nomenclatures of the variants are those reported by the Pango, Outbreak.info, and Stanford database websites.

b NTD, N-terminal domain (amino acids [aa] 13 to 305); RDB, receptor binding domain (aa 319 to 541); S1/S2, the junction between subunits S1 and S2 (aa 542 to 690).

c The table reports only the mutations that have been shown to have an impact on viral infectivity, transmissibility, or immunogenicity in published studies found on PubMed or preprints on bioRxiv or medRxiv. NA, data are not available.

d Infectivity was evaluated in pseudotyped viruses and/or by structural analysis.

e Transmissibility was evaluated by molecular epidemiology-based studies and/or *in vivo* studies.

f Disease severity was evaluated by analyzing clinical outcomes in terms of long-lasting infections and/or hospitalization period.

**FIG 3:**
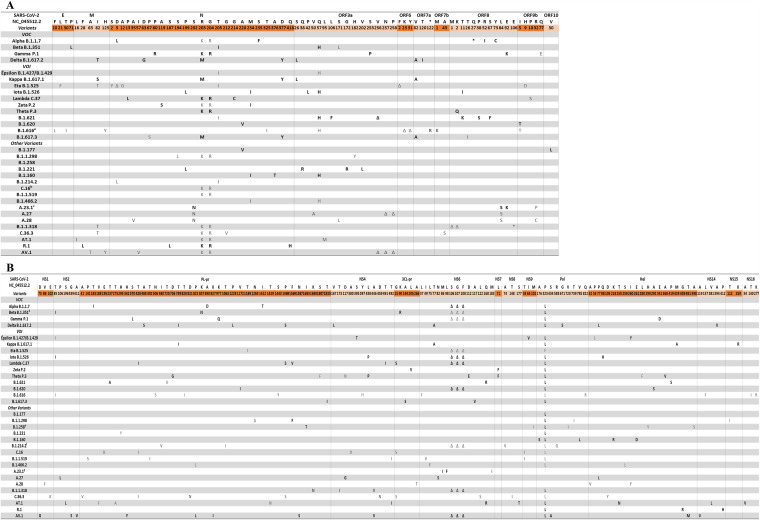
Mutations underlying the currently circulating variants in the SARS-CoV-2 proteins. Only mutated positions are reported. (A) The different structural and regulatory proteins are depicted. (B) The nonstructural proteins are depicted. The consensus sequence for each variant was defined as nonsynonymous substitutions or deletions that occurred in >75% of sequences within that lineage. Each mutation (such as P323L in the viral polymerase) is indicated by a first letter that is the symbol for the reference amino acid of NC_045512.2 (e.g., P), a number for the amino acid position in the wild-type protein (e.g., 323), and a second letter representing the amino acid actually found in the sequence analyzed (e.g., L). The nomenclatures of the VOCs and some of the VOIs were those reported by WHO and Pango, while the rest of the VOIs and other variants were reported by Pango. Mutations in black refer to the mutations reported by the Nextstrain, Outbreak.info, Pango lineages, and Stanford database websites, while mutations in gray are those that we identified by analyzing entire high-quality viral genome sequences from GISAID (*n* = 1,223,383). ^a^ A large deletion of 9 amino acids between residues 23 and 31 of ORF6 is detected in all sequences of this variant. ^b^ The mutations G238C in the nucleocapsid protein and G172C in the protein encoded by ORF3a are detected with prevalences of about 30.3% and 30.5%, respectively. ^c^ The mutations S2Y and R203K in the nucleocapsid protein are present with prevalences of about 12.1% and 13.4%, respectively. ^d^ The helicase mutation T588I is detected with a prevalence of about 21.9%. ^e^ The mutation E195D in NS6 is present with a prevalence of about 25.7%. ^f^ The mutation T51I in NS10 is present with a prevalence of about 56.1%. ^g^ The mutations L741F in PL-pro and T599I in Hel are present with prevalences of 6.2% and 2.1%, respectively.

Furthermore, SARS-CoV-2 variants are also characterized by mutations scattered throughout different proteins (Fig. 3). Notably, certain variants share mutations like S202N, R203K, G204R, T205I, M234I, and D377Y in the nucleocapsid protein, Q57H in the open reading frame 3a (ORF3a)-encoded regulatory protein, and L84S in the ORF8-encoded regulatory protein (Fig. 3A). Similarly, some mutations have been reported in nonstructural (NS) proteins, such as T85I in NS2, the S106-G107-F108 deletion in NS6 in 3 VOCs and 7 VOIs, and the well-known P323L in the viral polymerase in nearly all VOCs and VOIs (Fig. 3B). Interestingly, the use of specific multiplex quantitative PCRs (qPCRs) targeting specific mutations and/or deletions within the entire genome could be used for discrimination of VOCs (24).

Overall, although the exact roles of mutations scattered throughout different proteins still need to be elucidated, the fixation of these mutations in several VOCs and VOIs suggests they have importance in virus propagation and fitness. These mutations could play a role in hampering CD4^+^ and CD8^+^ T-cell responses, thus contributing to jeopardizing full SARS-CoV-2 immune control (25). These concepts support the evidence that extending sequencing from the spike to the full genome can provide more information for surveillance purposes and in regard to SARS-CoV-2 lineages, disease severity, and replication enhancement.

### Spike mutations localized in the N terminus (amino acids [aa] 13 to 305).

S13I was only detected in the Epsilon VOI and has been reported to reduce susceptibility to several NTD-targeting monoclonal antibodies, suggesting a potential role in immune escape (26).

L18F was detected in the Gamma P.1 VOC and A.27 variant. This mutation has been shown to potentially confer resistance to neutralization by monoclonal antibodies (27). Previously, it was a characteristic mutation of the Beta B.1.351 VOC also. Currently, its prevalence has decreased to about 38%, and it is mainly detected in two sublineages (B.1.351.2 and B.1.351.3) with prevalences of more than 90%.

T20N was only detected in the Gamma P.1 VOC. This mutation has been shown to potentially confer resistance to neutralization by monoclonal antibodies (28).

D80A and D80G were detected in the Beta B.1.351 VOC and AV.1 variant. These mutations have been shown to confer different degrees of resistance to neutralizing antibodies targeting the N terminus of the spike protein, suggesting that their potential role is to act as immune escape mutations (27, 29, 30).

W152C, W152L, and W152R were detected in the Epsilon VOI, R.1 variant, and C.36.3 variant, respectively. W152C has been reported to reduce susceptibility to several NTD-binding monoclonal antibodies, again suggesting a potential role in immune escape (26).

D215G was detected in the Beta B.1.351 VOC, B.1.616 VOI, and AT.1 variant. This mutation has been shown to cause partial resistance to neutralization (27).

A222V has been a typical genetic marker of the B.1.177 variant since it emerged in Spain and then spread throughout Europe during the summer of 2020. This mutation did not alter SARS-CoV-2 infectivity and antigenicity. A study has shown that all neutralizing NTD monoclonal antibodies bind efficiently to A222V (29).

D253G was detected in the Iota B.1.526 VOI. A recent study has suggested the potential capability of this mutation to reduce binding to monoclonal neutralizing antibodies (29).

### Spike mutations localized in the RBD (aa 319 to 541).

V367F occurs in the A.23.1 variant and is localized in a specific epitope recognized by neutralizing antibodies (31, 32), and it has been reported to slightly reduce neutralization by antibodies (33). Furthermore, in an *in silico* study, V367F increased the binding affinity to human ACE2 (34), which was associated with a modest increase in viral infectivity (23).

K417N and K417T characterize the Beta B.1.351 and the Gamma P.1 VOCs, respectively. Although they reduce the binding affinity of the receptor binding domain with ACE2 (35, 36), these mutations could also act as immune escape mutations (30, 37, 38). In particular, K417N and K417T can confer resistance to therapeutic monoclonal antibodies and to convalescent-phase sera of recovered patients (30, 37). Regarding the vaccine-elicited monoclonal antibodies, recent studies have shown that K417N or K417T, in combination with E484K and/or N501Y, can reduce the efficiency of neutralization (30, 38). Furthermore, K417N was selected when recombinant vesicular stomatitis virus expressing the SARS-CoV-2 spike was cultured in the presence of vaccine-elicited antibodies (30). Recently, it has also been detected in Delta B.1.617.2 VOC sublineages (AY.1 and AY.2).

N439K was detected in the B.1.258, B.1.466.2, and AV.1 variants. The presence of this mutation has been associated with slightly higher viral loads in nasopharyngeal swab samples than were found for the wild-type strain, consistent with increased infectivity (39). Notably, a recent study has suggested that N439K can confer resistance against several monoclonal neutralizing antibodies and can reduce the activity of convalescent-phase sera from individuals recovered from infection (39). However, it is noteworthy that this reduction in susceptibility applied to only a small number of plasma samples and was much smaller than the reductions observed with other variants.

L452R characterizes the Delta B.1.617.2 VOC and the Epsilon B.1.427/B.1.429 and Kappa B.1.617.1 and B.1.617.3 VOIs. It was also detected in other variants, including C.16, A.27, and C36.3. This mutation has been associated with a modest increase in SARS-CoV-2 infectivity as measured by soluble mACE2 (40). Interestingly, L452R (alone or in combination with other mutations, such as A475V, V483A, and F490L) may favor viral escape from specific monoclonal antibodies, as well as from convalescent-phase sera of recovered individuals (32). Recently, L452Q has been reported in the Lambda C.37 VOI, with an impact similar to that of L452R (41).

Y453F was detected in the B.1.1.298 variant, briefly sustaining a cluster of human infections that arose initially in minks in the summer of 2020. By structural analysis, this mutation has been associated with a potential increase in binding affinity to ACE2 (42). Furthermore, preliminary evidence suggests that Y453F may also act as an immune escape mutation conferring reduced susceptibility to monoclonal antibodies (37).

S477N was originally detected in the B.1.160 variant circulating in Portugal and was recently identified in the B.1.620 VOI and a sublineage of the Iota B.1.526 VOI. S477N is present in published sequences with a frequency of 2.6% (3) as of 9 July 2021. An early study has shown that S477N increases viral infectivity through enhanced interactions with ACE2 (43). Furthermore, this mutation is located in an epitope that is targeted by a large variety of monoclonal antibodies. The role of this mutation in affecting the neutralization efficiency of vaccine-elicited antibodies is still controversial, even if some studies showed no reduction in susceptibility to antibodies (30, 40).

T478K was originally identified in the Delta B.1.617.2 VOC and recently in the B.1.1.519 variant. The acquisition of the positively charged lysine (K) in the RBD has been proposed to increase the binding affinity of the spike glycoprotein for ACE2 (44). This mutation has been reported to confer neutralization to a sole monoclonal antibody (45).

V483A was reported in a recently identified B.1.616 VOI and is located in specific epitopes recognized by several neutralizing antibodies. By structural analysis, this mutation has been associated with partial resistance to monoclonal antibodies (31).

E484K, which was first identified in the Beta B.1.351 and Gamma P.1 VOCs, is increasingly being reported in other variants, including the Alpha B.1.1.7 VOC and the following VOIs: Eta B.1.525, Zeta P.2, Theta P.3, B.1.621, and B.1.620, as well as the B.1.1.318, AT.1, R.1, and AV.1 variants. Furthermore, E484K characterizes around 6% of the more recently identified A.23.1 variant (23). Position 484 resides in a dominant neutralizing epitope where mutations usually have the largest effect on binding to neutralizing antibodies (46). In line with this concept, different studies have shown that E484K (including as a single mutation) can favor viral escape from a large variety of monoclonal antibodies and from convalescent-phase sera of recovered individuals (40, 47, 48). Notably, the Alpha B.1.1.7 VOC with E484K showed a 50% reduction in neutralization efficiency by monoclonal antibodies targeting RDB (49). Interestingly, E484K was rarely found together with L452R in any variant (including VOCs or VOIs), with a total prevalence of <0.003% of all sequences in GISAID as of 29 July 2021 (3). Notably, it has been associated with a small but significant reduction in viral neutralization by vaccine-elicited monoclonal antibodies (30). Additionally, E484K was reported to have emerged after 73 days of coincubation with a highly neutralizing plasma from a recovered patient (47). The emergence of E484K was followed by the acquisition of an insertion in the N terminus of the spike glycoprotein conferring full resistance to plasma neutralization (47). Finally, E484K has been detected in individuals undergoing SARS-CoV-2 reinfection, particularly when detected in the genetic backbone of the Beta B.1.351 VOC (10).

E484Q has recently been reported in the newly emerged Kappa B.1.617.1 and B.1.617.3 VOIs. This mutation can abrogate an electrostatic interaction between the spike residue E484 and the ACE2 residue K31 (50). Its role in modulating viral infectivity and antigenicity still needs to be elucidated. Similarly to E484K, E484Q was associated with a 10-fold decrease in neutralization efficiency by vaccine-induced antibodies, suggesting a role (albeit moderate) as an immune escape mutation (51). No synergistic reduction in neutralization efficiency was observed when E484Q was combined with L452R (51).

N501Y is a critical genetic marker for the 3 first VOCs identified, Alpha B.1.1.7, Beta B.1.351, and Gamma P.1 (36, 52, 53). Recently, it has also been detected in the Theta P.3 and B.1.621 VOIs, as well as in the A.27 and A.28 variants. This mutation is located in the tip of the receptor binding domain and can increase the binding affinity for ACE2 (35, 46, 54), thus contributing to an increase in SARS-CoV-2 infectivity as shown in a murine model (53). The enhanced binding affinity can be explained by the fact that the presence of this mutation can favor the establishment of new interactions with ACE2 (in particular hydrogen bonds with ACE2 residues 41 and 353) and can induce a more open conformation of the RBD (54–56). At the same time, it has been postulated that such an increased binding affinity for ACE2 can also reduce the probability of interaction with antibodies targeting the RBD, partially contributing to mechanisms underlying SARS-CoV-2 immune evasion (57). The copresence of N501Y along with other mutations in the Beta and Gamma VOCs has been shown to confer complete resistance to neutralization by several monoclonal antibodies targeting the RBD, as well as reduced neutralization or complete resistance to neutralization by convalescent-phase sera of recovered individuals (30). Notably, a recent study highlighted the emergence of N501Y (along with other mutations) in an immunosuppressed patient with a long-lasting infection (58).

### Synergistic effect of mutations in NTD and RBD, characterizing the currently identified VOCs, on spike antigenicity.

The copresence of mutations in the NTD and RBD can play a critical role in mechanisms underlying evasion of neutralizing antibodies by SARS-CoV-2. This is the case for the Beta B.1.351 VOC, characterized by 2 point mutations (D80A and D215G) and a deletion of 3 residues in the NTD and 3 key mutations in the RDB (K417N, E484K, and N501Y) (Fig. 1). Different studies have highlighted that the Beta B.1.351 VOC can reduce the efficiency of neutralization by multiple antibodies targeting the NTD and RBD that are elicited by both natural infection and vaccination (27, 59–64). In particular, the Beta B.1.351 VOC has also been associated with an 11- to 33-fold decrease in neutralization efficiency by convalescent-phase sera and a 3.4- to 8.5-fold decrease in neutralization by vaccine-induced antibodies (27, 48, 60, 62–65). It is worthy of mention that such variants can be transmitted particularly in individuals developing low antibody titers, thus playing a role in reinfection or hampering the effectiveness of the current vaccine campaigns. In this regard, a recent study has compared the neutralizing titers of 58 convalescent-phase sera collected at the time of primary infection and after 9 months (64). This study showed that after 9 months, convalescent-phase sera had a mean 6-fold reduction in neutralizing titer and 40% of them lacked any neutralizing activity against Beta B.1.351 (64).

A similar scenario is observed for the combination of mutations in the Gamma P.1 VOC. This variant is characterized by 5 point mutations in the NTD (L18F, T20N, P26S, D138Y, and R190S) and 3 in the RBD (K417T, E484K, and N501Y) (Fig. 1). This variant can escape antibody neutralization, even if to a lesser extent than that observed for the Beta B.1.351 VOC. This can be explained by the lack of deletions in the NTD. In particular, the Gamma P.1 VOC was associated with decreases in neutralizing activity ranging from 6.5- to 13-fold for convalescent plasma and from 2.2- to 2.8-fold for vaccine-induced antibodies.

Notably, the combinations of NTD and RBD mutations characterizing the B.1.351 and P.1 VOCs confer partial or full resistance to the monoclonal antibodies casirivimab and bamlanivimab, respectively, used for the treatment of individuals infected with SARS-CoV-2 (11), again reinforcing the role of such combinations of mutations in mediating SARS-CoV-2 evasion of neutralizing antibodies.

Only moderate resistance to neutralization by convalescent-phase sera (4- to 6.7-fold) or by vaccine-induced antibodies (2- to 2.9-fold) was observed for the 2 sublineages (B.1.427 and B.1.429) of the Epsilon variant, characterized by more limited accumulation of mutations in the NTD and RBD: L452R in the RBD of both sublineages and the NTD mutations S13I and W152C only in the sublineage B.1.429 (Fig. 1) (59, 66). A comparable scenario has been highlighted for the Alpha B.1.1.7 VOC, characterized by a single RBD mutation (N501Y) coupled with 2 deletions in the NTD (Del 69/70 and Del 144) (Fig. 1). In particular, this variant was associated with reduced neutralizing activity by convalescent-phase sera (3-fold reduction) and vaccine-induced antibodies (2-fold) (22, 27, 57, 67, 68). This modest decrease in neutralizing efficiency is mainly mediated by the deletion at position 144, which is capable of affecting binding to most antibodies targeting the NTD (22, 27, 69).

Finally, the recently identified Delta B.1.617.2 VOC is characterized by a substantial accumulation of critical point mutations in the NTD and RBD (T19R, G142D, L452R, and T478K), coupled with a deletion at positions 157 and 158. Such a combination of mutations may be responsible for the 60% increase in viral transmissibility (compared to that of the Alpha B.1.1.7 VOC) (70) and for the decrease in neutralization efficiency observed for this VOC. In this regard, a very recent study has shown that the Delta B.1.617.2 VOC was resistant to neutralization by some monoclonal antibodies targeting the NTD and RBD, such as bamlanivimab. Furthermore, convalescent-phase sera, collected up to 12 months postinfection, were 4-fold less potent against this VOC than against the Alpha B.1.1.7 VOC. Notably, neutralizing antibodies isolated from individuals who had received one dose of the Pfizer or AstraZeneca vaccine barely inhibited Delta B.1.617.2 VOC. Conversely, two doses of the above-mentioned vaccines generated a neutralizing response against Delta B.1.617.2 VOC in 95% of individuals that was only 3- to 5-fold lower than that against the Alpha B.1.1.7 VOC (64). These findings support the crucial role of full-dose vaccination in controlling the spread of the virus. At the same time, this supports that partial immunity at the population level may fuel viral evolution and facilitate the selection of immune escape variants.

### Spike mutations localized in the junction domain between subunits S1 and S2 (aa 542 to 690).

Mutations in the junction domain between subunits S1 and S2 are not perceived to directly alter spike antigenicity. Nevertheless, they can induce long-term rearrangements in the RBD or have an impact in the fusion process, thus playing a potential role in enhancing viral infectivity.

D614G was originally detected in clade 20A, which emerged during the early phases of the pandemic. So far, G (glycine) has replaced the D (aspartate), becoming the wild-type amino acid at position 614 in all the SARS-CoV-2 clades circulating worldwide (13). A recent study has shown that D614G can increase viral infectivity on human lung cells or cells expressing bat or pangolin ACE2, presumably by shifting the conformation of the spike protein toward a fusion-competent state, in line with other studies supporting an increase in human-to-human viral transmissibility (13, 71–74).

Furthermore, D614G can abolish a hydrogen bond interaction with T859 of a neighboring monomer, thus destabilizing the spike trimer and in turn increasing the interaction with ACE2, further reinforcing the role of this mutation in enhancing SARS-CoV-2’s infectivity and, in turn, its transmissibility (13). This concept is in keeping with *in vivo* studies highlighting an association of D614G with higher viral loads in the upper respiratory tract of SARS-CoV-2-infected patients, as well as with a younger age of patients (72, 73).

So far there is no clear evidence that D614G may alter the efficacy of the current treatments that are based on monoclonal antibodies or convalescent-phase sera or that of the vaccine strategies in use to tackle SARS-CoV-2 circulation.

Q677H, detected in the Eta B.1.525 VOI and the C.36.3 variant, is localized in proximity to the furin cleavage site (aa 682 to 685). There is evidence that this mutation could cooperate with the other mutations contributing to the enhanced infectivity observed for this variant (75).

P681H and P681R have been detected in the Alpha B.1.1.7 and Delta B.1.617.2 VOCs and in the Kappa B.1.617.1, Theta P.3, B.1.617.3, B.1.621, and B.1.620 VOIs, as well as in the B.1.1.519, A.23.1, B.1.1.318, and AV.1 variants. They are adjacent to the furin cleavage site (23, 76). *In vitro* studies have shown that P681H can favor cleavage by the cellular furin protease, thus resulting in enhanced fusion activity of the SARS-CoV-2 spike protein (76). Based on a recent *in silico* study, these mutations also reside within predicted B- and T-cell epitopes (25, 77). The role of these mutations in affecting antibody neutralization is still under investigation. Furthermore, controversial results are available on their capability of interfering with cytotoxic immune response (25, 78). Indeed, so far, only one study has reported that P681H slightly alters recognition of specific CD8^+^ T cell epitopes, potentially weakening the CD8^+^ T-cell-mediated immune response (25).

### Deletions in the spike glycoprotein.

Del H69/V70 is a deletion at positions 69 and 70 that characterizes the Alpha B.1.1.7 VOC and was also detected in viral strains that sustained the SARS-CoV-2 outbreak in minks in Denmark and other European countries during the summer of 2020 (79, 80). Recently, it has been detected in the Eta B.1.525 VOI, the B.1.620 VOI, and the B.1.1.298, A.28, and C.36.3 variants. A recent *in vitro* study has shown that the copresence of Del H69/V70 with N501Y can increase SARS-CoV-2’s infectivity (81). Notably, this deletion was also observed in immunosuppressed patients following treatment with convalescent plasma, suggesting that it is capable of conferring reduced susceptibility to neutralizing antibodies (79, 82). In *in vitro* experiments, the deletion H69/V70 had 2-fold-higher infectivity than the wild type and could rescue the reduced infectivity in the presence of the spike mutation D796H (79). This deletion also reduced viral susceptibility to specific monoclonal antibodies targeting epitopes in the NTD (79). It has also impaired PCR diagnosis, since this deletion can abrogate detection of the S gene by some currently available real-time (RT)-PCR assays (83).

Del Y144 is a deletion at position 144 that is characteristic of the Alpha B.1.1.7 VOC and was also detected in the Eta B.1.525 and the B.1.620 and B.1.616 VOIs and in the B.1.1.318 and AV.1 variants. This deletion is located within the repeated deletion region (RDR) (aa 138 to 145) that composes a large immune-dominant B-cell epitope. A recent study has shown that the presence of this deletion in combination with other point mutations can confer resistance to some NTD-binding monoclonal antibodies, suggesting a potential role as an immune escape mutation (82). Additionally, the deletion in RDR has been detected in the recently identified Theta P.3 VOI.

Del L242-L244 is a deletion encompassing 3 positions, L242, A243, and L244, that characterizes the Beta B.1.351 VOC, Theta P.3 VOI, and B.1.620 VOI. This deletion is located within a large immune-dominant B-cell epitope recognized by the monoclonal antibody 4A8. Its presence confers resistance to this antibody, supporting its role as an immune escape mutation (82).

### Other recently identified spike mutations with uncertain function.

H66D, G142V, and G669S occur in a recently identified B.1.616 VOI. G142V is located within the RDR (aa 138 to 145) that composes a large immune-dominant B-cell epitope as mentioned above. Recently, G142D has been detected in the Delta B.1.617.2 VOC and AV.1 variant. However, the role of these mutations still needs to be elucidated.

S98F was detected in the B.1.221 variant and is located in the N-terminal domain of subunit S1. No information is available on the biological or clinical effects of this mutation.

E154K was recently reported in the Kappa B.1.617.1 VOI. It is located in the N-terminal domain, specifically, in an epitope recognized by neutralizing antibodies, even if its role in affecting the recognition of this epitope has not yet been defined (32).

F157L and Q613H occur in the A.23.1 variant. They are localized in the N terminus and in the junction domain, respectively. Furthermore, F157L is localized in specific epitopes recognized by neutralizing antibodies (31, 32). Similarly to D614G, it has been postulated that Q613H can contribute to enhancing SARS-CoV-2’s infectivity by favoring the cleavage of the spike glycoprotein and in turn promoting the fusion between the viral envelope and cell membrane (23).

R346K and R346S were detected in the B.1.621 VOI and the C.36.3 variant. This position is located in the receptor binding domain and is in an epitope recognized by monoclonal antibodies. There is initial evidence that this mutation may induce escape from monoclonal antibodies (31).

Q414K and N450K were recently identified in a B.1.214.2 variant, and both reside in the RBD. Notably, Q414K is located close to K417 and, therefore, is part of an epitope recognized by antibodies (84). N450K is involved in the RBD/ACE2 interaction and has been proposed to increase ACE2 binding (84) and to reduce susceptibility to several monoclonal antibodies (40).

F490S was recently identified in the Lambda C.37 VOI and has been associated with escape from monoclonal antibodies (administered singly or in combination) and convalescent-phase sera (33, 41).

I692V was detected in viral strains that sustained the SARS-CoV-2 outbreak in minks in Denmark and other European countries (80). This mutation is localized in the junction region between the S1 and S2 domains of the spike glycoprotein downstream from the furin cleavage site (80).

### Conclusions.

The spike glycoprotein is undergoing a constant process of genetic diversification that has led so far to the identification of a large variety of point mutations and deletions underlying the currently identified SARS-CoV-2 variants. Although experiments based on infection models are limited, there is clear evidence that spike mutations, particularly in the receptor binding domain, play a crucial role in modulating SARS-CoV-2’s infectivity and antigenicity. Overall, this reinforces the need for ongoing molecular surveillance programs to guide the development and usage of vaccines and of therapeutics based on monoclonal antibodies and convalescent-phase sera. At the same time, the increasing circulation of variants with immune escape mutations supports the need to periodically update the formulation of the current vaccines and to test the efficacy of monoclonal antibodies in clinical use against newly arising variants in order to avoid potential loss of clinical efficacy.

## MATERIALS AND METHODS

The mutations and the variants cited in this review are those that were highlighted by at least one among the GISAID (https://www.gisaid.org/), Nextstrain (https://nextstrain.org/), Outbreak.info (https://outbreak.info/), Pango (https://cov-lineages.org/), and Stanford (https://covdb.stanford.edu/) database websites (3, 18–21) and that have been associated with any clinical or diagnostic impact according to published manuscripts present on PubMed or preprint manuscripts on bioRxiv or medRxiv. Based on these criteria, 32 currently circulating variants have been included (Table 2). Among them, 4 were defined as VOCs and 11 as VOIs by definitions provided by the WHO, CDC, or ECDC (Table 2). The remaining 17 variants have not yet been classified as VOCs or VOIs, although they harbor mutations associated with increased viral infectivity, transmissibility, and altered antigenicity. Variants detected only in the first phases of the pandemic and not circulating any longer have not been included.

**TABLE 2:** Nomenclatures of variants

SARS-CoV-2 variant	Origin of identification	No. of SARS-CoV-2 sequences (*n* = 1,223,383)^*a*^
Variants of concern (VOCs)^*b*^		
20I/501Y.V1^*c*^ or B.1.1.7^*d*^^,^^*e*^^,^^*f*^ or Alpha^*h*^	English	790,848
20H/501Y.V2^*c*^ or B.1.351^*d*^^,^^*e*^^,^^*f*^ or Beta^*h*^	South African	16,530
20J/501Y.V3^*c*^ or P.1^*d*^^,^^*e*^^,^^*f*^ or Gamma^*h*^	Brazilian	34,722
B.1.617.2^*d*^^,^^*e*^^,^^*f*^ or Delta^*h*^	Indian	103,539

Variants of interest (VOIs)^*b*^		
CAL.20C^*c*^ or B.1.427/B.1.429^*d*^ or Epsilon^*h*^	USA	32,715
B.1.617.1^*d*^^,^^*e*^^,^^*f*^ or Kappa^*h*^	Indian	3,435
B.1.525^*d*^^,^^*e*^^,^^*f*^ or Eta^*h*^	English/Nigerian^*i*^	2,312
B.1.526^*d*^^,^^*e*^^,^^*f*^ or Iota^*h*^	USA	28,057
C.37^*d*^^,^^*e*^^,^^*f*^ or Lambda^*h*^	Peru	220
P.2^*d*^^,^^*f*^ or B.1.1.28^*e*^ or Zeta^*h*^	Brazilian	3,733
P3^*d*^ or PHL-B.1.1.28^*d*^ or Theta^*h*^	Philippines	126
B.1.621^*d*^^,^^*e*^	Colombian	540
B.1.620^*d*^^,^^*e*^	Cameroonian	106
B.1.616^*e*^	French	38
B.1.617.3^*d*^^,^^*e*^^,^^*f*^	Indian	121

Other variants		
20E. EU1^*c*^ or B.1.177^*c*^^,^^*e*^	Spanish	132,246
B.1.1.298^*d*^^,^^*e*^ or Mink cluster V^*g*^	Danish	957
20A/S:439K^*c*^ or B.1.258^*d*^^,^^*e*^	Scottish	10,822
20A/S:98F^*c*^ or B.1.221^*c*^^,^^*e*^	Belgian	10,963
20A.EU2^*c*^ or B.1.160^*c*^^,^^*e*^	Portuguese	20,727
B.1.214.2^*c*^	Belgian	631
C.16^*c*^	Portuguese	585
B.1.1.519^*c*^	Mexicans	16,475
B.1.466.2^*c*^	Indonesian	799
A.23.1^*e*^	Ugandan	936
A.27^*c*^^,^^*d*^	German	256
A.28^*c*^	French	296
B.1.1.318^*c*^	English	535
C.36.3^*c*^	Egyptian	943
AT.1^*c*^	Russian	113
R.1^*c*^^,^^*d*^	Japanese	8,918
AV.1^*c*^^,^^*d*^	English	139

a Number of SARS-CoV-2 sequences retrieved from GISAID (until 7 July 2021) and analyzed in the study.

b Considered a VOC or VOI by the WHO/CDC/ECDC.

c The nomenclature was reported by the Nextstrain website.

d The nomenclature was reported from the Stanford database, according to the Pango lineages.

e The nomenclature was reported from the Pango website, according to the Pango lineages.

f The nomenclature was reported from the Outbreak.info website, according to the Pango lineages.

g The nomenclature was reported from the GISAID website.

h The nomenclature reported from the World Health Organization (WHO).

i Detected for the first time in the United Kingdom and currently named the Nigerian variant.

SARS-CoV-2 genome sequences (*n* = 1,223,383 as of 7 July 2021) were retrieved from the GISAID database (3) and used to accurately define the specific pattern of mutations for each variant (Table 2). Stringent quality filters were applied in order to include only entire sequences characterized by high quality (identified as genomes of >29,000 nucleotides, with the presence of <1% ambiguous nucleotides and <0.05% unique amino acid mutations, and with no insertions or deletions unless verified in the sequence by the submitter).

Sequences were aligned by Bioedit using the NC_045512.2 SARS-Cov-2-Wuhan-Hu-1 isolate as the reference sequence. The alignment of sequences containing no nucleotide ambiguities was imported into and analyzed in the Nextclade tool in order to define mutations and to reveal which variant they belong to, while Seqscape software was used for sequences containing nucleotide ambiguities. Mutations were defined as amino acid substitutions using the NC_045512.2 SARS-Cov-2-Wuhan-Hu-1 isolate as the reference sequence. Sequences having a mixture of wild-type and mutant residues at single positions were considered to have the mutant(s) at that position.

Residues characterizing the 4 VOCs were also mapped on the three-dimensional structure of the SARS-CoV-2 spike protein (Fig. 2), using the methodology reported in the supplemental material.
