# Supplementary material for: SARS-CoV-2 Variants and Their Relevant Mutational Profiles: Update Summer 2021
Source: Microbiol Spectr. 2021 Nov 17;9(3):e01096-21. doi: 10.1128/Spectrum.01096-21 (PMC8597642; doi:10.1128/Spectrum.01096-21)
Supplement: SUPPLEMENTAL FILE 1 — Supplemental material. Download SPECTRUM01096-21_Supp_1_seq10.pdf, PDF file, 0.01 MB [file spectrum01096-21_supp_1_seq10.pdf]

Methods

Model generation and preparation

We used the SARS-CoV-2 spike protein and P17 fab complex with one RBD in close state deposited in the Protein Data Bank (1) with the PDB code 7CWL (2). For our computational analysis the P17 fab chains were removed.

By using the spike glycoprotein sequence, obtained from the UniProt database (3) sequence (P0DTC2) as template, we generated the spike model by means of the Prime homology modeling tool (Prime, Schrödinger, LLC, New York, NY, 2018). The 6XRA (4) PDB model was adopted to generate the S2 domain of spike protein. By means of the Maestro suite (Schrödinger Release 2021-1: Maestro, Schrödinger, LLC, New York, NY, 2021), we added the S2 domain to our generated model, thus obtaining the spike protein structure.

The model was prepared by using the Protein Preparation Wizard tool (Protein Preparation Wizard; Schrödinger, LLC, New York, NY, 2018; Prime, Schrödinger, LLC, New York, NY, 2018) implemented in Maestro using OLPS-2005 (5) as force field. Residual crystallographic buffer components and water molecules were removed, missing side chains were built using the Prime module, hydrogen atoms were added, side chains protonation states at pH 7.4 were assigned and an energy minimization simulation was performed.

All figures were generated by means of PyMOL tool (PyMOL Molecular Graphics System, Version 2.0 Schrödinger, LL).

References

1. Berman HM. 2000. The Protein Data Bank. Nucleic Acids Research 28.

2. Yao H, Sun Y, Deng Y-Q, Wang N, Tan Y, Zhang N-N, Li X-F, Kong C, Xu Y-P, Chen Q, Cao T-S, Zhao H, Yan X, Cao L, Lv Z, Zhu D, Feng R, Wu N, Zhang W, Hu Y, Chen K, Zhang R-R, Lv Q, Sun S, Zhou Y, Yan R, Yang G, Sun X, Liu C, Lu X, Cheng L, Qiu H, Huang X-Y, Weng T, Shi D, Jiang W, Shao J, Wang L, Zhang J, Jiang T, Lang G, Qin C-F, Li

L, Wang X. 2021. Rational development of a human antibody cocktail that deploys multiple functions to confer Pan-SARS-CoVs protection. Cell Research 31.

3. Bateman A, Martin M-J, Orchard S, Magrane M, Agivetova R, Ahmad S, Alpi E, Bowler-Barnett EH, Britto R, Bursteinas B, Bye-A-Jee H, Coetzee R, Cukura A, da Silva A, Denny P, Dogan T, Ebenezer T, Fan J, Castro LG, Garmiri P, Georghiou G, Gonzales L, Hatton-Ellis E, Hussein A, Ignatchenko A, Insana G, Ishtiaq R, Jokinen P, Joshi V, Jyothi D, Lock A, Lopez R, Luciani A, Luo J, Lussi Y, MacDougall A, Madeira F, Mahmoudy M, Menchi M, Mishra A, Moulang K, Nightingale A, Oliveira CS, Pundir S, Qi G, Raj S, Rice D, Lopez MR, Saidi R, Sampson J, Sawford T, Speretta E, Turner E, Tyagi N, Vasudev P, Volynkin V, Warner K, Watkins X, Zaru R, Zellner H, Bridge A, Poux S, Redaschi N, Aimo L, Argoud-Puy G, Auchincloss A, Axelsen K, Bansal P, Baratin D, Blatter M-C, Bolleman J, Boutet E, Breuza L, Casals-Casas C, de Castro E, Echioukh KC, Coudert E, Cuche B, Doche M, Dornevil D, Estreicher A, Famiglietti ML, Feuermann M, Gasteiger E, Gehant S, Gerritsen V, Gos A, Gruaz-Gumowski N, Hinz U, Hulo C, Hyka-Nouspikel N, Jungo F, Keller G, Kerhornou A, Lara V, le Mercier P, Lieberherr D, Lombardot T, Martin X, Masson P, Morgat A, Neto TB, Paesano S, Pedruzzi I, Pilbout S, Pourcel L, Pozzato M, Pruess M, Rivoire C, Sigrist C, Sonesson K, Stutz A, Sundaram S, Tognolli M, Verbregue L, Wu CH, Arighi CN, Arminski L, Chen C, Chen Y, Garavelli JS, Huang H, Laiho K, McGarvey P, Natale DA, Ross K, Vinayaka CR, Wang Q, Wang Y, Yeh L-S, Zhang J, Ruch P, Teodoro D. 2021. UniProt: the universal protein knowledgebase in 2021. Nucleic Acids Research 49.
4. Cai Y, Zhang J, Xiao T, Peng H, Sterling SM, Walsh RM, Rawson S, Rits-Volloch S, Chen B. 2020. Distinct conformational states of SARS-CoV-2 spike protein. Science 369.
5. Jorgensen WL, Maxwell DS, Tirado-Rives J. 1996. Development and Testing of the OPLS All-Atom Force Field on Conformational Energetics and Properties of Organic Liquids. Journal of the American Chemical Society 118.
